# ‘Positive’ inter‐ictal clinical signs of functional neurological disorders are found in patients with functional dissociative seizures

**DOI:** 10.1111/ene.16430

**Published:** 2024-08-03

**Authors:** Margaux Cheval, Arnaud Lapostolle, Astrid De Liège, Louise Tyvaert, Charlotte Joly, Béatrice Garcin

**Affiliations:** ^1^ Neurology Department, Hopital Avicenne, Assistance Publique Hôpitaux de Paris Paris France; ^2^ Epileptology Unit, Reference Center for Rare Epilepsies, Department of Neurology, AP‐HP Pitié‐Salpêtrière Hospital Paris France; ^3^ Reference Center for Rare Epilepsies, Neurology Department University Hospital of Nancy Nancy France; ^4^ UPMC UMRS 1127, Inserm U 1127, CNRS UMR 7225, Institut du cerveau et de la moelle épinière (ICM) Paris France

**Keywords:** clinical examination, FND, functional dissociative seizures, Hoover's sign

## Abstract

**Background and purpose:**

Prior studies highlighted the high diagnostic specificity (ranging from 92% to 100%) of clinical signs observed in functional neurological disorders (FNDs). However, these signs are rarely looked for by epileptologists when trying to distinguish between functional dissociative seizure (FDS) and epileptic seizure. The aim of this study was to determine the prevalence of inter‐ictal clinical signs of FND in a cohort of patients with probable FDS. The secondary objective was to compare the prevalence of inter‐ictal FND clinical signs in FDS patients with age‐ and gender‐matched epileptic patients without FDS.

**Methods:**

Patients diagnosed with FDS seen at two tertiary care centres and epileptic outpatients were included in the study. Each patient underwent a physical examination, searching for inter‐ictal clinical signs of FND.

**Results:**

In the FDS group, 79% of patients presented at least one sign of FND, compared to 16.6% of patients with epilepsy (*p* < 0.001). Moreover, 66.6% of FDS patients presented three or more FND signs, whereas only 4.1% of epileptic patients did (*p* < 0.001). The median number of FND clinical signs in the FDS group was four (SD 1.7; 5.5). Using the threshold of three signs or more, the specificity of detecting three or more FND signs was 83.3%, with a sensitivity of 79.2%.

**Conclusion:**

Inter‐ictal clinical signs of FND are present in patients with FDS and should be looked for during neurological examination.

## INTRODUCTION

Functional dissociative seizure (FDS) is a common condition [1, 2, 3], responsible for significant disability and major healthcare costs [4]. Patients suffering from FDS endure prolonged delays in diagnosis [5], resulting in increased visits to emergency departments, consultations, additional investigations and social burdens [4, 6, 7, 8]. However, it has been shown that a clear diagnosis, combined with a properly conducted diagnostic explanation, leads to an improvement or cessation of FDS and is cost effective [6]. Providing new rapid diagnostic tools for FDS is therefore crucial. The diagnosis of FDS currently relies on patient and witness interviews, video recordings of seizures, as well as normal magnetic resonance imaging (MRI) and electroencephalography (EEG) [9]. Access to video‐EEG recordings is often limited and is usually reserved for complex cases. The diagnostic challenge also extends to other functional neurological disorders (FNDs), and several studies have been conducted previously to assess the diagnostic value of positive signs observed during the examination of patients with FND [10, 11]. These signs demonstrate a high diagnostic specificity for FND, ranging between 92% and 100% [11, 12]. However, epileptologists often overlook these signs when trying to distinguish between FDSs and epileptic seizures.

The primary objective of this study was to assess the prevalence of inter‐ictal clinical signs of FND in a group of patients diagnosed with probable FDS (International League Against Epilepsy definition) [9]. The secondary objective was to compare the occurrence of inter‐ictal FND signs between patients with FDSs and those with epileptic seizures without FDSs, to determine whether the presence of these clinical signs could contribute to the differential diagnosis of FDS.

## MATERIALS AND METHODS

### Patient selection

Patients diagnosed with FDS were prospectively included in the study after an evaluation in one of the two centres: a tertiary care hospital specialized in FND and another with expertise in epilepsy (each consecutive outpatient). Patients were excluded if FDS was clearly associated with other FNDs. In other words, they were not included if they had a spontaneous motor or sensory complaint or if they had a diagnosis of motor or sensory functional disorder. However, during a focused interview, some patients mentioned other functional symptoms that were not central to their main complaint. Diagnosis of FDS was established by clinicians, based on clinical history, witness‐provided video recordings of the manifestations, as well as results from MRI and EEG examinations [9]. Patients with probable FDS as defined by LaFrance et al. [9] were included. A few patients were diagnosed with FDS using video‐EEG before being included in the study (prolonged or standard video‐EEG recording, demonstrating normal seizure without concomitant EEG changes). Patients' evaluations were conducted during a single consultation. Patients in the FDS group were eligible for inclusion even if they were also suffering from comorbid epilepsy.

Patients with epilepsy were included in the study following outpatient evaluation at a tertiary care hospital specialized in epilepsy. Exclusion criteria were the association of epilepsy with other clearly identified FNDs or FDSs. In the epilepsy group, patients were followed up and all had either recorded seizures or clear epileptic anomalies on EEG recordings, concordant with the semiology of the seizures. Patients within the epilepsy group were matched for age and sex with those in the FDS group. The study was approved by the local ethics committee.

### Recorded variables

For the FDS group, medical history recording included the age at onset of FDS, the duration of the disease, any association with epilepsy, potential prescription of anti‐seizure medications, confirmation of the diagnosis with video‐EEG and any relevant additional medical history. The semiology of FDS was defined according to the classification of semiology published by Hubsch et al. [13]. For the epilepsy group, data collected included the age at onset of epilepsy and the duration and classification of epilepsy. In both groups, each patient underwent a physical examination to search for signs of FND [11, 12] (for more information on how to perform this clinical examination see Data S1). Patients were examined by two practitioners with FND experience (B.G. and M.C.) and sign positivity was defined as previously described in the literature [12].

**Table:** 

Checklist of positive clinical signs of FND collected during examination (Daum et al. [11], Aybek and Perez [12]) (for more information on how to perform this clinical examination see Data S1)
Validated Walking Hesitant or cautious walking Non‐economic posture Sudden knee buckling Fall toward support Dragging monoplegic leg Motor Collapsing/give‐away weakness Co‐contraction of antagonist muscles Spinal injury test Chair test Excessive slowness Hoover's sign Abductor sign Abductor finger sign Drift without pronation Superior limb flexion/extension sign (Hoover equivalent) Sensory Midline splitting Systematic failure during sensory testing Splitting of vibration Other/movement disorders Oculomotor complaint (i.e., excessive blinking, effortful facial expression during eye movement examination)/eye movement abnormalities during examination Distractibility Entrainment effect Not validated ‘Whack a mole’ sign Tremor frequency fluctuations Fixed dystonia

### Statistical analysis

Data are presented as mean (SD) for continuous variables and as count (percentages) for categorical variables. A comparison between the two groups was conducted using the Wilcoxon–Mann–Whitney test for numerical variables and Fisher's exact test for categorical variables. Statistical analyses were performed using R 4.1.2. The anonymized data supporting the findings of this study are available on request from the corresponding author.

## RESULTS

Twenty‐four patients with FDS and 24 age‐ and sex‐matched patients with epilepsy were included. Table 1 shows the comparison between the two groups. Nineteen out of 24 patients with FDS (79%) presented at least one sign of FND, whereas four out of 24 (16.6%) patients with epilepsy did (*p* < 0.001). Additionally, 17 out of 24 patients with FDS (70.8%) exhibited three or more signs of FND, whereas only one out of 24 patients with epilepsy (4.1%) did (*p* < 0.001). The median number of FND clinical signs in the FDS group was four (1.7; 5.2). Interestingly, one patient with FDS did not exhibit any signs during the initial assessment but did so at a 4‐month follow‐up. Amongst the four epileptic patients who showed signs of FND, two had comorbid functional somatic disorders (fibromyalgia, one; persistent postural‐perceptual dizziness, one) and a third had mild mental retardation (epileptic encephalopathy). Amongst the patients with epilepsy and FDS (*n* = 7), three had idiopathic generalized epilepsy and four had focal epilepsy.

**TABLE 1:** Comparison between patients with FDS and those with epilepsy but without FDS.

	Patients with FDS (*N* = 24)	Patients with epilepsy without FDS (*N* = 24)	*p* value^a^ corrected
Gender (female)	19 (79%)	19 (79%)	(matched)
Age (years)	34 (23; 50)	32 (23; 51)	(matched)
Association with epilepsy (yes)	7 (29.1%)	–	–
Age at onset of epilepsy or FDS (years)	31 (20; 42)	17.5 (14. 32)	–
Disease duration (years)	5 (3; 7.5)	10 (5; 17)	–
FDS confirmed with VEEG (yes)	6 (25%)	–	–
ASD prescriptions (yes)	10 (41.6%)	24 (100%)	–
Type of epilepsy	–		–
Generalized idiopathic	3/7	9 (37.5%)	–
Epileptic encephalopathy	–	0	–
Focal	*4/7*	13 (54.1%)	–
Focal temporal	*3/7*	6 (25%)	–
Focal other localization	–	9 (37.5%)	–
FDS semiology^b^		–	–
Hypermotor	16 (66.6%)	–	–
Akinetic	6 (25%)	–	–
Pseudo‐syncope	5 (20.8%)	–	–
Axial dystonic	1 (4.1%)	–	–
Dystonic motor	4 (16.6%)	–	–
Association with other functional somatic disorders			–
Fibromyalgia	3 (12.5%)	1 (4.1%)	–
Respiratory	0	0	–
Urinary	4 (16.6%)	0	–
PPPD (without functional gait)	2 (8.3%)	1 (4.1%)	–
Digestive	4 (16.6%)	0	–
Post‐COVID symptoms	2 2 (8.3%)	0	
Number of FND signs	4 (1.7; 5.5)	Median 0 (0; 0), mean 0.41	*p* = 0. 003
Presence of at least one FND sign (yes)	19 (79%)	4 (16.6%)	*p* < 0. 001
Presence of more than one FND sign (yes)	18 (75%)	3 (12.5%)	*p* < 0. 001
Presence of 3 or more FND signs (yes)	16 (66.6%)	1 (4.1%)	*p* < 0. 001
Presence of FND signs (yes)			
Walking			
Hesitant or cautious walking	4 (16.6%)	2 (8.3%)	NS
Non‐economic posture	2 (8.3%)	0	NS
Sudden knee buckling	0	0	NA
Fall toward support	1 (4.1%)	0	NS
Dragging monoplegic leg	1 (4.1%)	0	NS
Motor			
Collapsing/give‐away weakness	14 (58.3%)	2 (8.3%)	*p* < 0. 001
Co‐contraction of antagonist muscles	0	0	NA
Spinal injury test	3 (12.5%)	0	NS
Chair test	1 (4.1%)	0	NS
Excessive slowness	4 (16.6%)	1 (4.1%)	NS
Hoover's sign	10 (41.6%)	0	*p* = 0.002
Abductor sign	7 (29.1%)	0	NS
Abductor finger sign	1 (4.1%)	0	NS
Drift without pronation	3 (12.5%)	0	NS
Superior limb flexion/extension sign (Hoover equivalent)	0	0	NA
Tremor with functional characteristic below	4 (16.6%)	*0*	–
Distractibility	4 (16.6%)	0	NS
Entrainment effect	3 (12.5%)	0	NS
‘Whack a mole’ sign	1 (4.1%)	0	NS
Frequency fluctuations	4 (16.6%)	0	NS
Sensitive			
Systematic failure	0	1 (4.1%)	NS
Midline splitting	8 (33.3%)	1 (4.1%)	NS
Splitting of vibration	5 (20.8%)	1 (4.1%)	NS
Other			
Oculomotor complaint/eye movement abnormalities during examination	10 (41.6%)	2 (8.3%)	*p* = 0.05
Fixed dystonia	0	0	NA

*Note*: Data are given as median (first quartile, third quartile) for continuous variables and as count (percentages) for categorical variables.

Abbreviations: ASD, antiseizure drug; COVID, coronavirus disease; FDS, functional dissociative seizure; FND, functional neurological disorder; PPPD, persistent postural‐perceptual dizziness; VEEG, video‐electroencephalography.

^a^ Wilcoxon–Mann–Whitney test was used to compare groups for numerical variables and Fisher's exact test for categorical variables. *p* values were corrected for multiple comparisons using the Benjamini–Hochberg method.

^b^ One patient could exhibit several types of FND with different semiology (total > 24).

**FIGURE 1:**
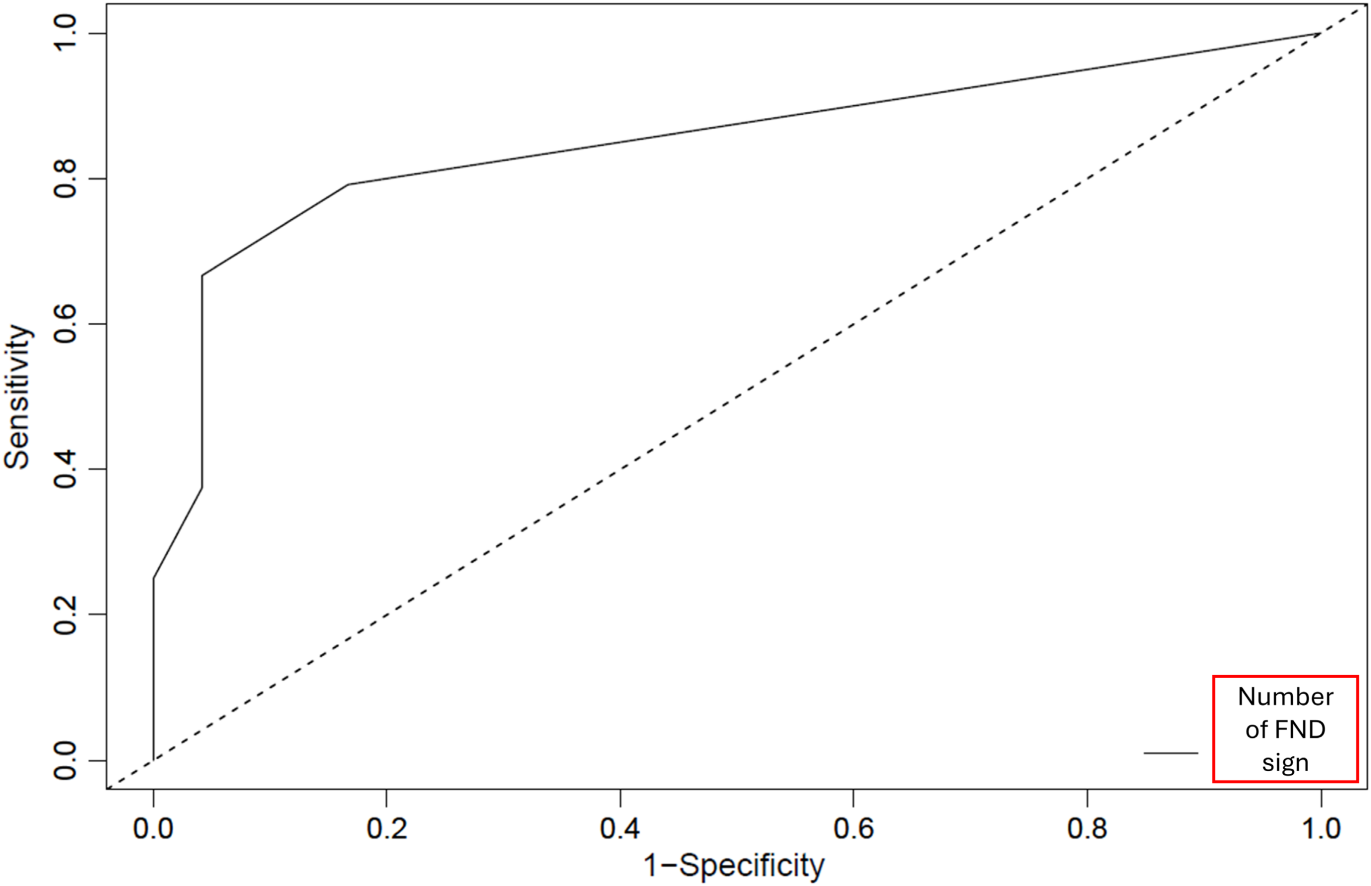
ROC curve of the number of FND signs for the diagnosis of FDS. Using the Youden index method, the optimal cutoff point was 3.

Figure 1 displays the receiver operating characteristic (ROC) curve for the number of FND signs for the diagnosis of FDS. Using the Youden index method, the optimal cutoff point was determined to be 3 (area under the curve 0.850). With a threshold of three signs or more, the specificity of detecting three or more FND signs was 83.3%, whilst the sensitivity was 79.2%.

During the interview, some patients mentioned additional functional symptoms that were not the main complaint (FDS group, gait anomalies, *n* = 4 patients; tremor, *n* = 2 patients; motor signs, *n* = 3 patients; epilepsy group, motor signs (walking) two patients). Two patients had a complaint of vertigo and instability when walking, but no gait disorder, and were thus categorized as having PPPD.

## DISCUSSION

This study is the first to show a significantly higher prevalence of FND signs in patients with FDS compared to epileptic patients without FDS. The identification of three or more FND signs demonstrates high specificity for the diagnosis of FDS. Specifically, the proportion of patients presenting with give‐away weakness, Hoover's sign and oculomotor complaints was significantly higher in the FDS group compared to the epilepsy group.

This study has several limits. First, patients with probable FDS were included, and most of the patients did not benefit from a video‐EEG recording. However, 25% had at least one EEG recording of FDS. Secondly, investigators were not blinded to diagnosis and clinical history and they may have been biased in their clinical assessment. Thirdly, the number of patients included in the study is relatively small. Indeed, replicating the current findings in a multicentric blinded study focusing solely on patients with confirmed FDS would be highly valuable.

There was a significantly higher prevalence of FND signs in patients with FDS, with a proportion of 79% of FDS patients showing at least one sign at clinical examination. It is important to highlight that those patients had no sensory‐motor complaint and that only the clinical examination could reveal these signs. Until now, the diagnosis of FDS has predominantly relied on clinical signs observed during ictal manifestations [12]. Incorporating these signs, observed outside of episodes, into routine neurological evaluations could facilitate faster diagnoses of FDS. Motor signs and midline splitting/splitting of vibration were the most frequent FND signs found in FDS patients. These signs are easy to look for and can be integrated into the basic neurological examination during an initial evaluation. Patients with FDS were examined in the same way as all other patients with FND, with the exception that all the signs on the checklist were carefully searched for. It was estimated that it takes no more than 10 extra minutes to search for these signs during a classic neurological examination. One of the limitations of this study is that examiners were not blinded to the diagnosis of FDS or epilepsy and may therefore have been more inclined to validate positive signs in the FDS group. However, the investigators all had specific experience of the clinical examination of FND. It might be more difficult for a neurologist who is not trained in FND to identify those clinical signs. This highlights the need for more training in FND during medical studies [14].

The presence of positive signs of FND in these patients underscores the shared overlap between motor/sensory FND and FDS. It suggests common vulnerability and underlying mechanisms in FDS and other FND presentations [2, 15]. It would be interesting to assess a larger sample of patients to see whether there was a specific association between the semiology of the FDS and the clinical signs at examination.

It is important to note that some patients with epilepsy also exhibited signs of FND. Therefore, it is crucial not to rely solely on the presence of a single sign, but to consider the association of multiple signs for an accurate diagnosis. The coexistence of FDS and epilepsy is common, and whether these clinical signs could help identify those patients at risk of having both types of seizures is hypothesized. Through the ROC curve analysis, it was found that the presence of three signs was the most discriminative value between the two groups. Most epileptic patients with signs of FND had a comorbid condition such as depressive syndrome or mild mental retardation, which are known factors in the development of FNDs [15]. Some had other non‐neurological functional disorders.

## CONCLUSIONS

Positive clinical signs of FND are present in patients with FDS and should be looked for during inter‐ictal neurological examination. Epileptologists are still unfamiliar with these clinical signs, and education on this semiology is crucial. In this small cohort, preliminary evidence was provided of the relevance of these signs to discriminate between FDSs or epileptic seizures. Association of several of these signs (three or more) seems to be the most reliable indicator.

## AUTHOR CONTRIBUTIONS

**Margaux Cheval:** Conceptualization; investigation; methodology; writing – original draft; formal analysis; data curation. **Arnaud Lapostolle:** Investigation; writing – review and editing. **Astrid De Liège:** Investigation; writing – review and editing. **Louise Tyvaert:** Conceptualization; writing – review and editing. **Charlotte Joly:** Investigation; writing – review and editing. **Béatrice Garcin:** Conceptualization; investigation; writing – review and editing; methodology; validation; data curation; supervision; project administration.

## FUNDING INFORMATION

No funding to disclose.

## CONFLICT OF INTEREST STATEMENT

None of the authors has any conflict of interest to disclose.

## ETHICS STATEMENT

It is confirmed that the authors have read the Journal's position on issues involved in ethical publication and affirm that this report is consistent with those guidelines.

## INSTITUTIONAL REVIEW BOARD STATEMENT

The study was approved by the local ethics committee of Université Paris Nord. The patients signed an informed consent form for the use of medical reports and recordings data.

## Supporting information


Data S1.

## Data Availability

The data that support the findings of this study are available on request from the corresponding author. The data are not publicly available due to privacy or ethical restrictions.
