# Supplementary material for: ‘Positive’ inter‐ictal clinical signs of functional neurological disorders are found in patients with functional dissociative seizures
Source: Eur J Neurol. 2024 Aug 3;31(10):e16430. doi: 10.1111/ene.16430 (PMC11414800; doi:10.1111/ene.16430)
Supplement: Supplementary file 1 — Data S1. [file ENE-31-e16430-s001.docx]

**Supplementary material :**

**How-to : The Check-list of positive clinical sign of FND (based on review by Aybeck et al. 2022, Daum et al. 2014)** ^11,12^

|  | **How to test ?** |
| --- | --- |
| ***Walking*** | |
| Hesitant or cautious walking | *Look for disproportionate hesitation and caution in gait (contrasting with good balance, strength, sensation)* |
| Non-economic posture | *Look for postures during gait that require good balance and strength such as flexed knees* |
| Sudden knee buckling | *Look for sudden buckling of the knee, usually with each step. Extreme cases will show knee touching the floor at each step* |
| Fall towards support | *The patient tends to fall in the direction of support (wall, furniture)* |
| Dragging monoplegic leg | *The weak leg is “dragged” like a piece of wood/ inanimate object, without spastic circumduction, usually along the floor surface* |
| ***Motor*** | |
| Collapsing/ Give-away weakness | *When testing strength against resistance; initially good and then sudden loss of resistance from the patient* |
| Co-contraction of antagonist muscles | *When testing strength, no movement at the joint (elbow, for example) occurs because co-contraction of agonist and antagonist is observed* |
| Spinal injury test | *Passively put both legs in a flexed position, sole of feet touching the bed during a lying position; observe if the weak leg stays in this position (functional) or falls back on the bed (“organic”* |
| Chair test | *In case of severe gait disorder, ask the patient to propel a chair with wheels; movements of the legs will be better than during gait* |
| Excessive slowness | *Look for disproportionate slowness in gait (slow stepping movements contrasting with lack of limb bradykinesia)* |
| Hoover sign | *Ask to flex the healthy hip against resistance and observe/feel the strength of hip extension of the weak leg (if patient lying: examiner’s hand under the heel, if sitting under the thigh). Compare with voluntary hip extension of the weak leg: if involuntary strength >voluntary strength, Hoover is positive* |
| Abductor sign | *Ask to abduct both legs against resistance: observe/ feel if involuntary abduction of the weak leg occurs (functional) or not (“organic”)* |
| Abductor finger sign | *In severe unilateral hand weakness, ask the patient to abduct the fingers of the healthy hand against resistance; observe if involuntary abduction of the 5th finger in the weak hand occurs (functional) or not (“organic”)* |
| Drift without pronation | *Arms stretched out, palms up in a full supination position, fingers adducted, eyes closed for 10 s: if a downward drift is seen, observe if a movement of pronation also occurs* |
| Superior limb flexion/extension sign (Hoover equivalent) | *Arms flexed at 30°, forearms held near the wrists by examiner. Ask patient to flex the healthy arm against resistance and observe/feel if increased extension of the weak arm occurs (functional) or not (“organic”). Then ask patient to flex weak arm and observe if extension of healthy arm occurs (“organic”) or not (functional)* |
| *Tremor with functional characteristic below* | |
| Distractibility | *Pause during ballistic movement or during other motor/mental task or change in amplitude and frequency* |
| Entrainment effect | *Ask to imitate tapping motion with one hand and observe the change in tremor frequency on the other* |
| “Whack a mole” sign | *Immobilize the limb affected by tremor : the tremor appears in another body segment (head, trunk, other arm, or legs).* |
| Frequency fluctuations | *Observe changes in tremor frequency during history taking/ examination/arriving or leaving the examination room: periods of unexplained improvement/ disappearance of symptom* |
| ***Sensitive*** | |
| Systematic failure | *Patients always fails in a discriminative task (eg, pin or prick/cold-hot/upgoing or downgoing joint)* |
| Midline splitting | *Touch the trunk : the patient report exact splitting of sensation in the midline* |
| Spliting of vibration | *Place the tuning fork on a flat bone (sternum or frontal bone): patient report a difference in the sensation of the vibration over the left compared to the right side* |
| ***Other*** | |
| Oculomotor complaint / Eye movement abnormalities during examination | *Do a classical oculomotor exam : excessive blinking, effortful facial expression, increased latency, gaze deviation, limited range, absent frontalis contraction during upgaze or complaint about the difficulty of performing the requested test* |
| Fixed dystonia | *New onset dystonic posture without possibility to move the joint of the affected limb* |
